# Research on the unsteady flow characteristics of high specific speed axial flow impellers with small aspect ratio and double blades

**DOI:** 10.1038/s41598-025-85597-9

**Published:** 2025-03-10

**Authors:** Zhihui Lu, Fangming Zhou, Xiaofang Wang

**Affiliations:** 1 School of Mechanical Engineering, Liaoning Petrochemical University, Fushun, China; 2 School of Energy and Power Engineering, Dalian University of Technology, Dalian, China

**Keywords:** High-specific-speed axial impeller, Small aspect ratio, Tip clearance, Pressure fluctuation, Flow flied and vortices, Mechanical engineering, Physical oceanography

## Abstract

High specific speed impeller with low aspect ratio is ideal for high-speed pump-jet propulsion. This study investigates its unsteady hydrodynamic characteristics using experimental and numerical methods. At the design flow rate (Q_d_), the pressure amplitude at the blade’s leading and trailing edges varies significantly along the streamline. Blade shape minimally affects flow velocity but significantly impacts leading-edge pressure fluctuations. For the mainstream, the highest fluctuation peak occurs at 0.9Q_d_. The pressure amplitude gradually decreases from blade’s shroud to hub. Along circumferential direction the minimum pressure amplitude is located in the middle of the two blades. In gap flow, the leading-edge pressure peaks at Q_d_, with fluctuations primarily at 5 times and 10 times the rotation frequency. Meanwhile, pressure fluctuations in the tip clearance’s height direction exhibit a consistent distribution, reaching their maximum at the leading edge. Vortex structure analysis using various Q criteria reveals that increasing Q enhances vortexes in the impeller while reducing them in the diffuser. Moreover, flow rates result in a simultaneous decrease in vortexes within both components, while the pressure distribution on the isotropic vortex surface remained stable.

## Introduction

Pump jet, as a reliable power unit for navigational propulsion, has been widely used in high-specific-speed navigational vehicles due to its high propulsion efficiency and low noise^1^. Therefore, an increasing number of researches spring up on the performance of pump-jet propellers, involving various aspects and fields, among which the research on the tip clearance flow characteristics of pump-jet propeller is a hot spot.

After extensive and in-depth academic investigations, we have clarified that the tip clearance and internal flow field of the impeller have a significant effect on the rotating mechanical properties^2–4^. Numerous experimental and numerical studies^5,6^ have been devoted to revealing the tip clearance flow in pumps, especially the evolution of tip leakage vortices (TLV) and the flow mechanism in the clearance region. Scholars have conducted exhaustive studies on the performance of pump jet thruster^7^, efficiency loss mechanism^8^, cavitation phenomena^9^ and pressure pulsation characteristics^10,11^ for different clearance dimensions. Campos-Amezcua et al.^12^ explored the influence of the tip clearance of leaf on wheel-induced cavitation. Fanning et al.^13^ analysed the causes of inducer backflow, pointing out that blade inlet diffusion rather than tip clearance is a key factor affecting backflow. Kim et al.^14^ investigated the effects of cavitation and induced wheel gap size on pump performance and found that cavitation transferred from the induced wheel region likewise had a significant effect on pump performance. Kim et al.^15^, on the other hand, focused on the top of the blade gap flow and its effect on turbine performance and hydraulic efficiency. The study of leakage vortices and vertical cavitation vortices at the vane tips of axial pumps^16–18^. Combining numerical simulation and experimental analysis, M T Rahman et al.^19^ conducted in-depth research on the internal flow characteristics of centrifugal pumps, and revealed the location and intensity of vortices under different operating conditions by comparing simulation and experimental results. Ding et al.^20^, on the other hand, simulated and experimentally verified the internal flow characteristics of a centrifugal pump by adjusting the angle of vane outlet. The results showed that the hydraulic loss of the centrifugal pump and flow rate increased with the increase in the angle of vane outlet.

However, previous studies mostly focused on the conventional impellers with large aspect ratio^11,21,22^, and there are relatively few studies on the applications of impellers with small aspect ratio applied to high-specific-speed pump jets. This research focuses on the high-speed impeller with small aspect ratio, deeply analyses its flow characteristics, and explores the relationship between pressure fluctuation and the geometry, rotational speed, fluid flow characteristics and working conditions of the impeller. Taking the impeller with a clearance size of 0.5 mm as the research object and combining numerical and experimental methods, this paper examined the unsteady flow characteristics of high-specific-speed axial-flow impeller with small aspect ratio and studied the pressure fluctuation, flow field and vortex of new impellers, which provides a key basis for optimizing the performance of high-speed pump jets, improving propulsion efficiency and reducing noise.

## Numerical model

### Impeller with small aspect ratio

The performance parameters (S1 to S7) for conventional pump jets are shown in (Table 1). Different from previous study, this paper utilized an axial flow pump with a new type of impeller with a small aspect ratio, which is characterized by high rotational speed, high head, small flow rate, and high specific speed. Its performance parameters are shown in (Table 2). The specific speed n_s_ is defined as:

1 $$n_{s} = \frac{{3.56 \times n \times \sqrt Q }}{{H^{{\frac{3}{4}}} }}$$

The units of the variables in Eq. (1) are consistent with those shown in (Table 2).

**Table 1:** The performance parameters of pump jets available in the literature.

Model	Rotational speed (rpm)	Volume flow (m^3^/s)		Pump head (m)	Specific speed
S1	1000	0.009722		0.4	716
S2	1450	0.484		7.45	817
S3	280	47.789		24.26	646
S4	1450	0.8889		7	1159
S5	1450	0.345		6.44	769
S6	900	0.14		3.5	480
S7	1450	0.3903		7	768

**Table 2:** Parameters of pump.

Parameter	Value
Design flow rate Q_d_ (m^3^/s)	0.286
Design head H_d_ (m)	90
Rotational speed n (rpm)	17,500
Specific speed n_s_	1169
The number of blades of the inlet and outlet guide vanes Z_g_	5
Mean blade span (mm)	50

**Fig. 1:**
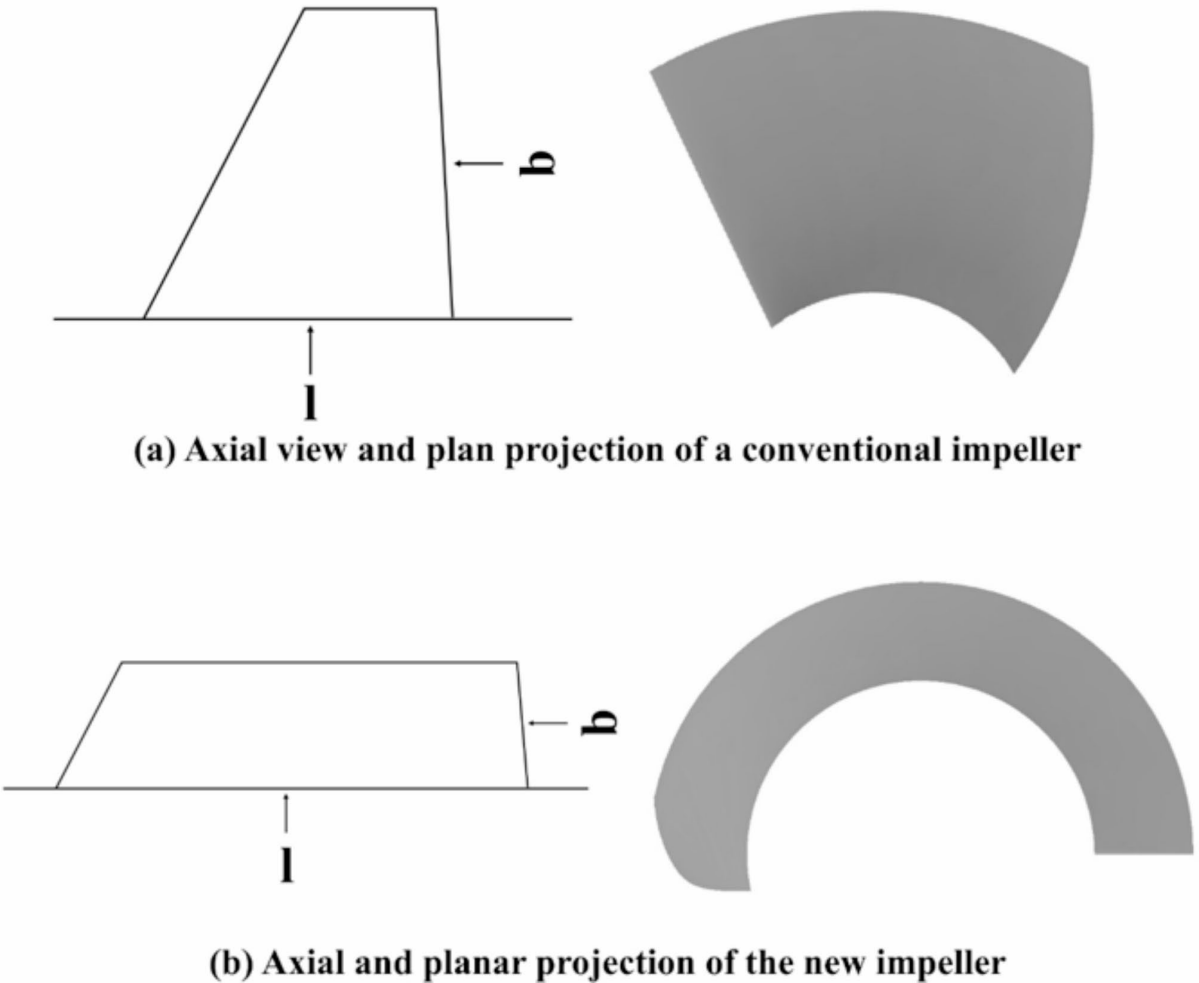
Diagram of conventional and new impellers.

Blade aspect ratio is an important parameter used to measure the shape characteristics of the blade, which refers to the ratio of the width of the blade (b) to the axial aspect length of the blade (l). The impeller of traditional pump jets has a large aspect ratio, whose axial plan and plane project diagrams are given in (Fig. 1a). However, this new type of impeller for high-specific-speed has a small aspect ratio, and its axial plan and plane projection diagram in (Fig. 1b). Meanwhile, this type of impeller has few blades, large wrap angle and long tip clearance.

### Physical modelling

**Fig. 2:**
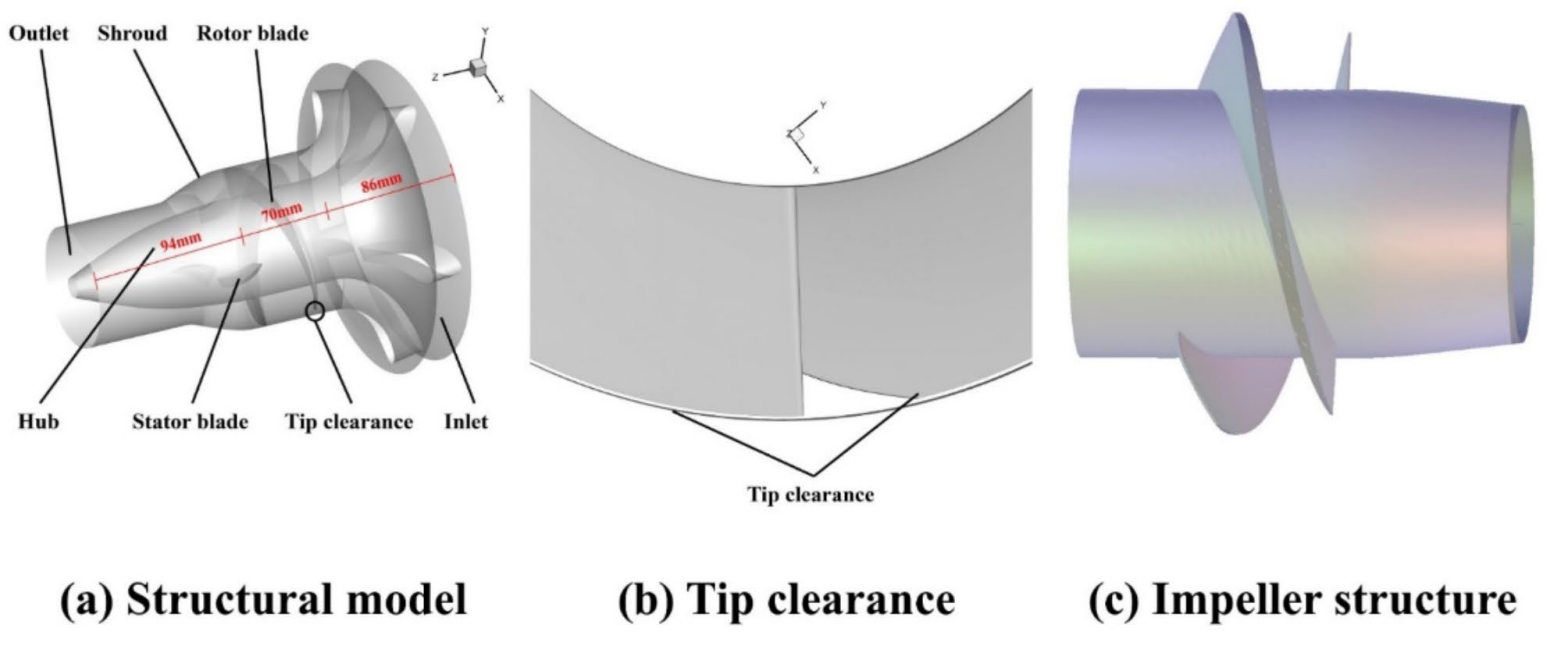
The structural model and tip clearance.

The structural model and tip clearance view of the new impeller are shown in (Fig. 2a,b). The impeller structure inside the pump is shown in (Fig. 2c). The pump was divided into three sections in the modelling process, where the lengths of the inlet, impeller and outlet sections were 86 mm, 70 mm and 94 mm, respectively. The cascade density was 1.8 and tip clearance (TC) was 0.5 mm, about 1% of the average span.

### Mesh

**Fig. 3:**
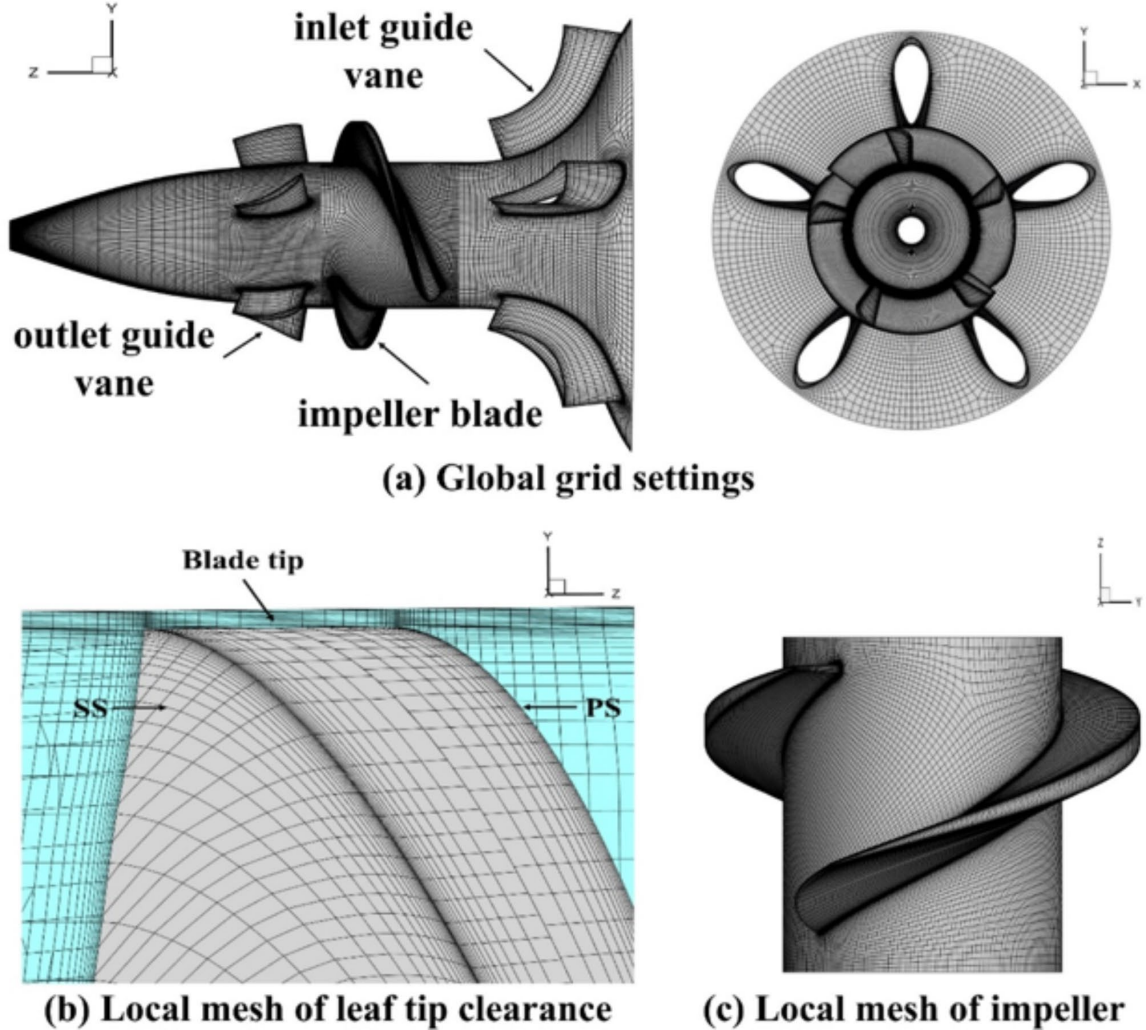
Grid setting for new axial pumps.

The computational domain inside the pump was meshed using a structured mesh in this paper, and the final global mesh obtained using a hexahedral mesh is shown in (Fig. 3a). In addition, to improve the accuracy of the study, the computational domain was divided into rotational and stationary domains which were encrypted with a local mesh grid. The rotating domain refers to the impeller, as shown in Fig. 3c, and the remaining domains are stationary sections. Considering the drastic change in the flow field near the tip of the impeller, a local mesh refinement scheme was used to generate the mesh at the tip of the impeller, as shown in (Fig. 3b). Meanwhile, in order to assess the influence of mesh density on the computational results, mesh independence validation was carried out, and four sets of meshes were used for the numerical simulation. The division results are shown in (Table 3).

**Table 3:** Simulation meshing.

Grid	Impeller	water intake	water outlet	Total number of grids
Case 1	1200505	163854	301526	1665885
Case 2	1350654	189562	367544	1907760
Case 3	1500408	212755	411200	2124363
Case 4	1650865	238421	456211	2345497

The actual head H and the theoretical head H_t_ of the pump can be calculated by:

2 $$H = \frac{{P_{{out}} - p_{{in}} }}{{\rho g}} + \left( {z_{{out}} - z_{{in}} } \right)$$

3 $$H_{t} = \frac{{M\omega }}{{\rho gQ}}$$

where* P*_*out*_ is the total pressure at the outlet;* P*_*in*_ is the total pressure at the inlet;* z*_*out*_ is the height of the outlet; * z*_*in*_ is the height of the inlet; M is the sum of the moments applied to the pressure surface of the blades, the suction surface, and the surfaces of the front and rear cover plates;* ω* is the rotational angular velocity of the impeller; Q is the volume flow rate of the pump.

The hydraulic efficiency of the pump can be calculated by:

4 $$\eta _{h} = \frac{H}{{H_{t} }}$$

As can be seen from Table 4, when the number of meshes was approximately 2.12 million, the calculation results tended to stabilize. After mesh independence verification, with the increase in the number of meshes, the key performance indicators of the impeller pump basically remained unchanged. In order to balance calculation accuracy and efficiency, the mesh in Case 3 was employed for subsequent calculations.

**Table 4:** Results calculated for different simulation grids.

q_m_/(kg/s)	Performance	Grid			
		Case1	Case2	Case3	Case4
269.59	Lift/(m)	140.26	142.05	143.15	143.15
	Efficiency/(%)	77.96	78.55	79.35	79.41
286.11	Lift/(m)	85.47	87.33	90.04	90.04
	Efficiency/(%)	68.56	69.05	70.75	70.82
300.25	Lift/(m)	57.34	51.33	46.95	46.95
	Efficiency/(%)	50.25	46.75	42.35	42.26

## Numerical setting

### Numerical methods

The fluid dynamics calculations were performed using CFX software. The SST k-ω turbulence model was used for steady state calculations. The boundary conditions were set as total pressure at the inlet, mass flow rate at the outlet and no slip wall at the wall. The convergence criterion was defined as: The computation was considered converged when the root mean square (RMS) residuals were below 1.0 × 10^− 6^. The advection scheme and turbulence numeric were set to high resolution. The coupling of the rotating and stationary domains, frozen rotor and transient rotor-stator methods were respectively applied to steady state and unsteady state simulations^11^. The time step was set to 2.86 × 10^− 5^ s for the transient simulation, equivalent to 1/120 T (T is the rotation period of the impeller), and the turbulence model of the RANS was used to obtain the flow structure and associated turbulence characteristics in the pump.

### Control equations

The shear stress transport (SST) turbulence model improves the simulation of turbulent boundary layer and separated flows by optimising the prediction of turbulent viscosity. The SST k-ω turbulence model has been widely used because of its excellent performance in predicting hydrodynamics and cavitation in pipeline propellers and pump-jet propellers^1,23–25^. Among them, Yongle et al.^25^ and Qin et al.^24^ comparatively analysed the effectiveness of the model in the hydrodynamic prediction of conduit propellers, and both confirmed that the SST k-ω turbulence model exhibited higher accuracy than the k-ε^26^ turbulence model family. In addition, Desheng Zhang’s team^27^ also found that the SST k-ω turbulence model had a smaller prediction error than the standard k-ω^28^ turbulence model when being used to investigate the effect of turbulence modelling for an axial pump. The SST k-ω turbulence model is constructed based on the k-ε turbulence model and the standard k-ω turbulence model. The two-equation turbulence model was subsequently further developed. Its governing equations include the Reynolds-averaged Navier-Stokes equation and the transport equations for turbulent kinetic energy and dissipation rate, which can be separately expressed as:

5 $$\frac{{\partial \left( {\rho U_{i} } \right)}}{{\partial t}} + \frac{{\partial \left( {\rho U_{i} U_{j} } \right)}}{{\partial x_{j} }} = - \frac{{\partial P}}{{\partial x_{i} }} + \frac{\partial }{{\partial x_{j} }}\left[ {\mu \frac{{\rho U_{i} }}{{\partial x_{j} }} + \rho \overline{{u_{i}^{\prime } u_{j}^{\prime } }} } \right]$$

6 $$\frac{{\partial \left( {\rho k} \right)}}{{\partial t}} + \frac{{\partial \left( {\rho u_{i} k} \right)}}{{\partial x_{i} }} = \frac{\partial }{{\partial x_{j} }}\left[ {\left( {\mu + \frac{{\mu _{t} }}{{\sigma _{k} }}} \right)\frac{{\partial k}}{{\partial x_{j} }}} \right] + P_{k} - \beta ^{ * } \rho \omega k$$

7 $$\frac{{\partial \left( {\rho \omega } \right)}}{{\partial t}} + \frac{{\partial \left( {\rho u_{i} \omega } \right)}}{{\partial x_{i} }} = \frac{\partial }{{\partial x_{j} }}\left[ {\left( {\mu + \frac{{\mu _{t} }}{{\sigma _{\omega } }}} \right)\frac{{\partial \omega }}{{\partial x_{j} }}} \right] + \frac{\omega }{{\sigma _{\omega } }}\frac{{\partial k}}{{\partial x_{j} }} - \beta \rho \omega ^{2}$$

where ρ is the density, U_i_ is velocity, P is pressure, µ is the dynamical viscosity, $\overline{{u_{i}^{\prime } u_{j}^{\prime } }}$ is Reynolds stress, k is the turbulent kinetic energy, ω is the turbulent dissipation rate, u_i_ is the velocity component, µ_t_ is the turbulent viscosity, P_k_ is the turbulent energy production rate, β* and β are the model parameters, and σ_k_ and σ_ω_ are model constants.

### Experimental validation

**Fig. 4:**
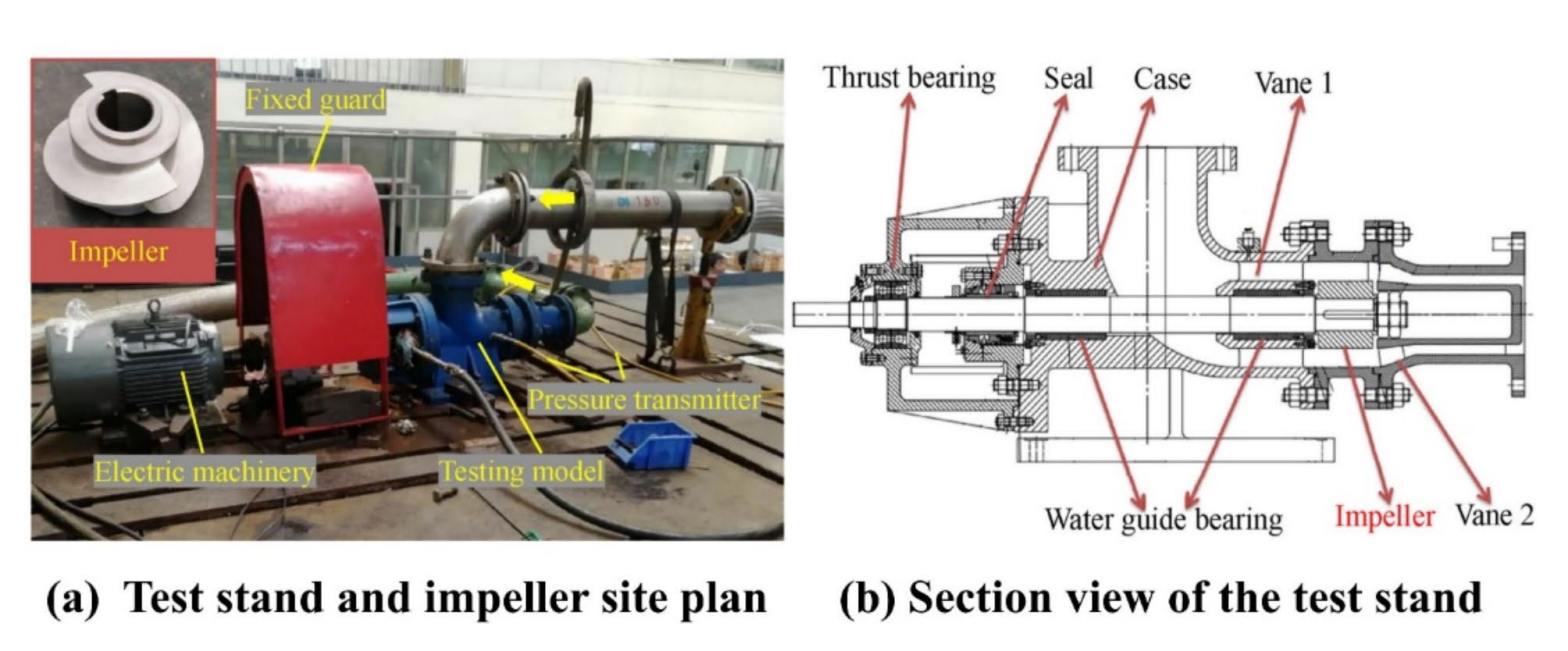
Test bed for validating the numerical methodology.

This paper selects the core hydraulic components composed of the impeller and the blades as the research object. Boundary conditions were established based on the conditions described in Sect. 2.1, and the corresponding test rig was constructed accordingly, as shown in (Fig. 4). The test section in Fig. 4b covers the area from the impeller inlet to the blade outlet, based on which the simulation domain was defined. Subsequently, numerical simulations were carried out for this simulation domain using a graphical approach.

**Fig. 5:**
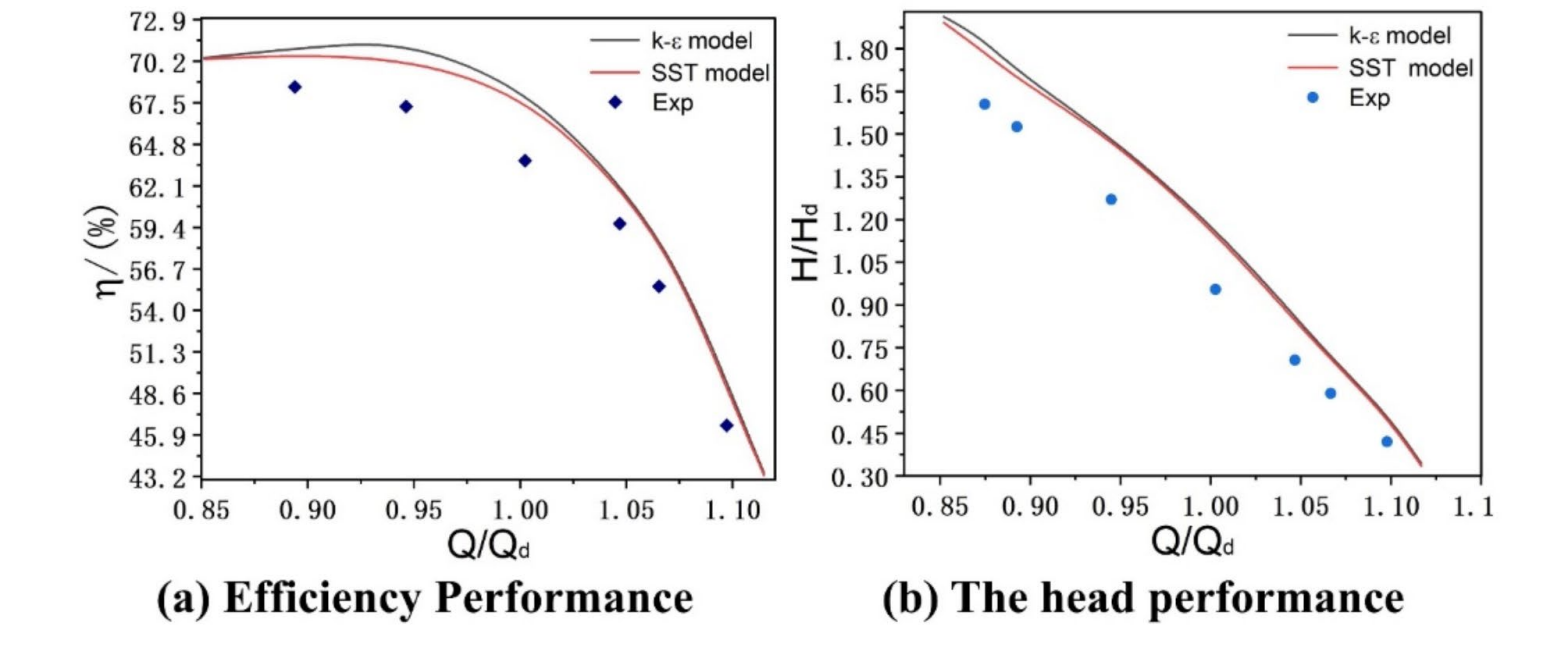
Performance comparison between simulation and experiment.

The SST turbulence model was used to compare with the standard k-ε model in the numerical simulation of the hydraulic model, and the results are shown in (Fig. 5). Meanwhile, the formula used to calculate the efficiency (η) is as:

8 $$\eta = \frac{{Q\rho gH}}{{\tau \omega }}$$

Where ω is the angular velocity; τ is the torque.

By analysing the simulation results, small discrepancy between the two models were observed. Figure 5 shows that SST model agrees better with experiments than standard k-ε. Therefore, the SST turbulence model was chosen for simulation in order to more accurately reflect the hydraulic properties under design conditions. Under the design conditions, the head and efficiency errors obtained from the SST model were less than 2.6 and 2.1%, respectively, which met the specified permissible criteria^29,30^, thereby implying the applicability and accuracy of the SST model in hydraulic modelling simulations. Figure 5 shows the efficiency and head by k-ε and k-ω.

### Setting

**Fig. 6:**
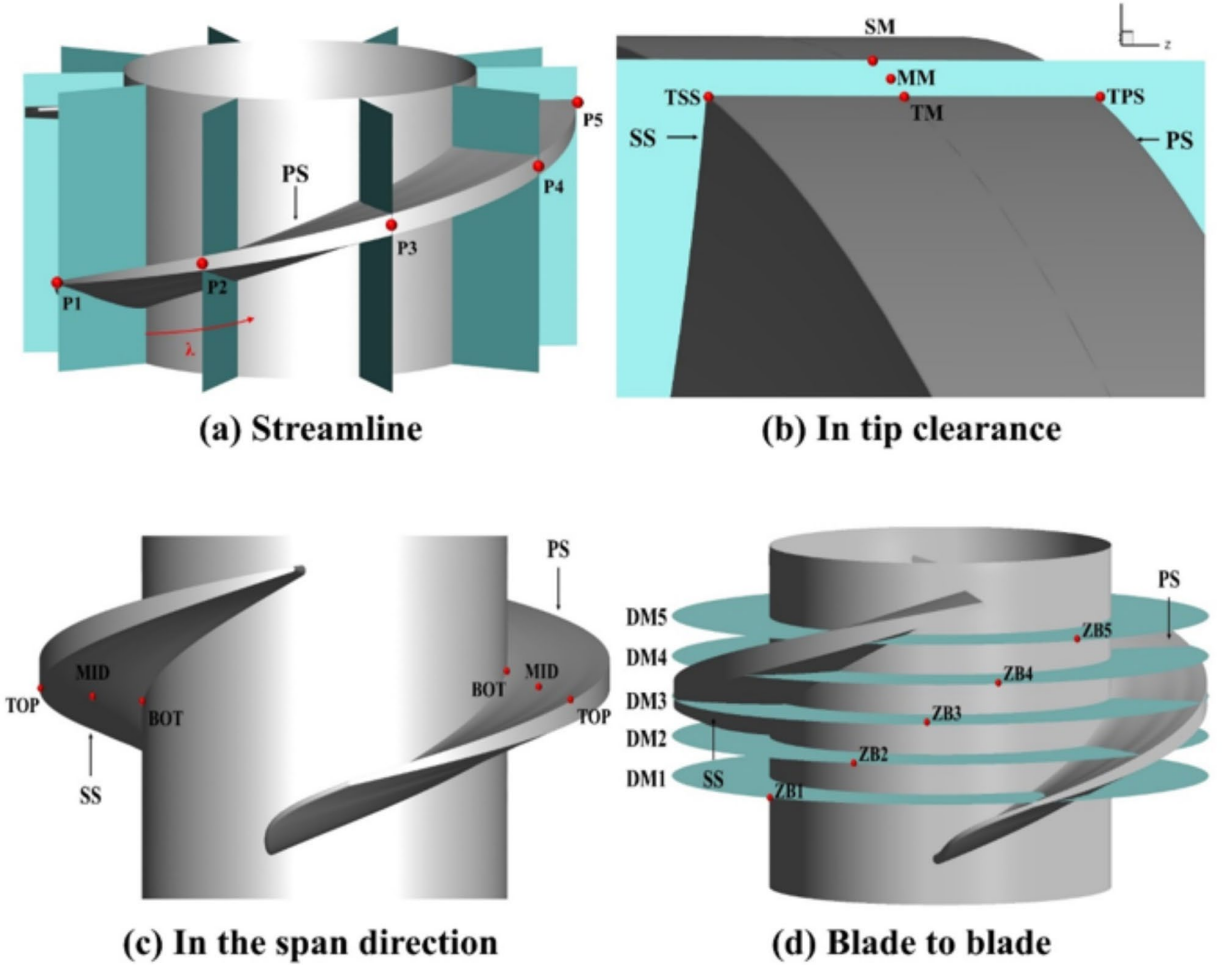
Setting of monitoring point.

With the aim of accurately capturing the flow field and pressure pulsation in the tip clearance of the impeller during unsteady flow simulations, we purposefully arranged several sets of monitoring points in the rotating domain. Figure 6 shows the location and profile schematic of these monitoring points in detail, where PS represents the pressure surface, SS represents the suction surface, and λ denotes the blade aspect ratio (i.e., the ratio of the length from the leading edge to the trailing edge of the blade). P1 to P5 denote five sets of axial profiles at the established monitoring points, which were equidistantly distributed in the circumferential direction. Figure 6b displays the axial profiles in the tip clearance, which shows the locations of five key points, including TPS (at the tip pressure surface), TSS (at the tip suction surface), TM (at the middle of the tip), SM (at the upper tip shroud), and MM (at the midpoint between TM and SM). The setting of these points helps us to have a more comprehensive understanding of the flow characteristics within the tip clearance of the impeller^31^. Figure 6c, on the other hand, shows the spreading positions of the monitoring points on the pressure surface (PS) and suction surface (SS), which are specifically classified as TOP (top of the blade), MID (middle of the top and bottom of the blade), and BOT (bottom of the blade). The monitoring points at these locations help us to gain insight into the pressure distribution and flow state at different sections of the blade. Figure 6d, clearly shows the location of the monitoring points at the inlet and outlet end faces (DM), which are essential for analysing the flow characteristics at the inlet and outlet of the pump.

In addition, in order to more comprehensively study the performance of the new impeller pump under different operating conditions, on the basis of the design flow rate Q_d_, an additional operating condition of 0.9 times Q_d_ and 1.1 times Q_d_ were also established for simulation. By comparing and analysing the simulation results under these working conditions, we gained a deeper understanding of the performance characteristics and flow law of the new impeller pump under various operating conditions.

## Result and discussion

### Pressure fluctuation

To facilitate the observation of the spectrogram, ΔP/P is used to represent the relative amplitude of the pressure pulsation, where ΔP is the amplitude of the pressure pulsation and P represents the designed average pressure; a variable f_r_ was utilized to represent the magnitude of the rotational speed frequency (Hz), which can be calculated by:

9 $$f_{r} = \frac{n}{{60}}$$

where n denotes the rotational speed (rpm).

#### Streamline pressure fluctuation

Due to the significant vibration and noise generated by the pump at a high speed, the pressure fluctuation in the main flow and gap flow of the impeller were analysed.

**Fig. 7:**
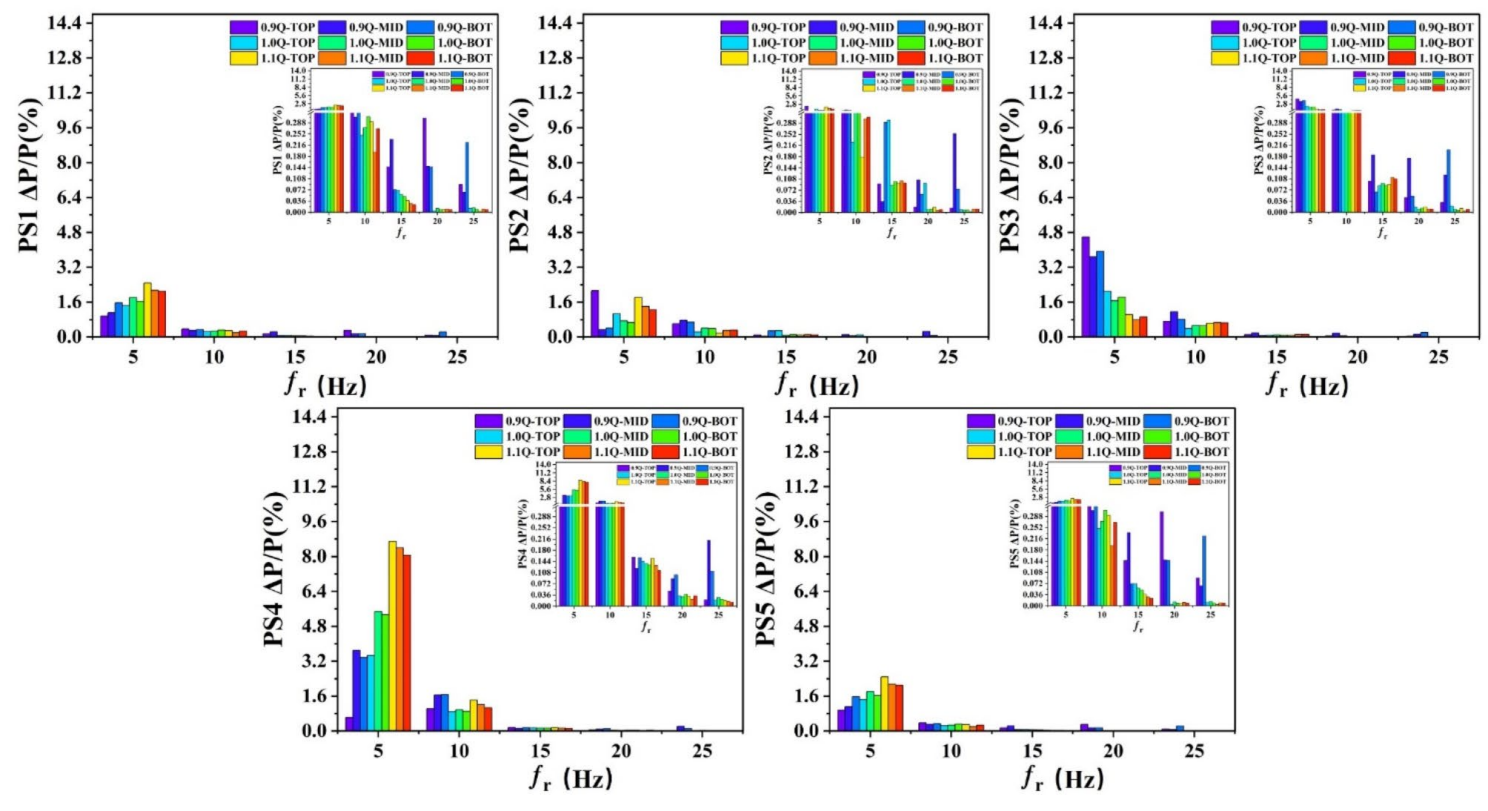
Spectra of pressure at different monitoring points on the pressure surface of impeller.

The pressure surface in the main flow field was firstly analysed. As depicted in Figure S1 and Fig. 7, the pressure on the pressure surface changed periodically under the three working conditions. Specifically, the pressure at 0.9Q_d_ was the largest, but the periodic curve trend was disorderly, indicating irregular pressure fluctuations, which further resulted in relatively large vibrations and noises. This was closely related to the geometry and rotational speed of the impeller, as well as fluid flow characteristics. For instance, irregularly shaped blades of the impeller might lead to uneven flow distribution, thereby resulting in pressure fluctuations. To facilitate the observation of the magnitudes of the values in the rotational frequency diagram, an enlarged view of the rotational frequencies from 10 to 25 in the original figure is attached as a subfigure in the figure. However, since the values after the rotational frequency of 10 are relatively small, their influence can be neglected. The focus is mainly on observing the patterns of the rotational frequencies of 5 and 10. Figure 7 shows the pressure spectra at different monitoring points on the pressure surface of the impeller under different working conditions. It can be seen that the peaks of pressure fluctuations at 5 and 10*f_r_ are mainly concentrated in the trailing edge area of the blade. This is attributed to the complex fluid flow in the trailing edge area, where unstable flows such as vortices and separations exist, resulting in large pressure fluctuations.

In the streamline direction, the pressure at the leading edge of the blade was the largest with relatively minor fluctuations because the leading edge was the first to be affected by the fluid, resulting in a relatively high pressure. Furthermore, the relatively small pressure fluctuations at the leading edge of the blade are caused by its relatively regular structure. The pressure value of the pressure surface initially decreased and then increased from the leading edge to the trailing edge of the blade, with the lowest pressure at the middle, which reflects the more energy loss at the middle due to the significant change in the fluid flow velocity, thus reducing pressure correspondingly. In the blade span direction, the pressure value progressively decreased from the blade tip to the hub, with pressure fluctuations concentrated at the leading edge of the blade near the blade tip. This is because the boundary conditions at the blade tip are different from the constraint conditions at the hub. The blade tip is relatively more open, and the fluid flow is less restricted. Under the action of the pressure difference, leakage vortices are prone to occur. Such leakage vortices can cause local changes in velocity and pressure, thereby generating pressure fluctuations. The large velocity gradient at the blade tip promotes the generation of turbulence. The coupling of velocity, pressure, and the turbulence structure exacerbates the instability of the flow field, making the pressure fluctuation more pronounced.

**Fig. 8:**
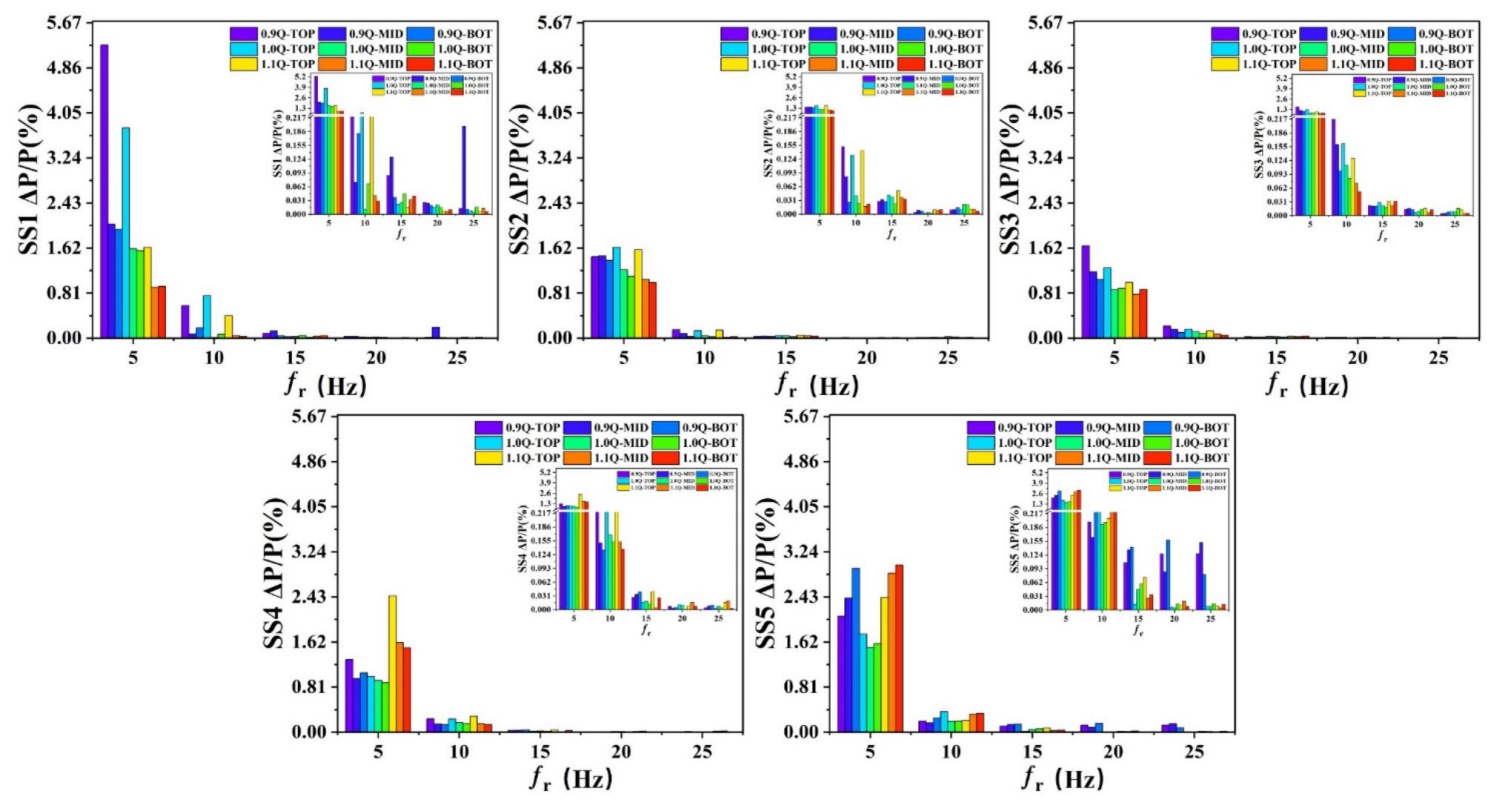
Spectra of pressure at different monitoring points on the suction surface of impeller.

Then the suction surface was analysed. As shown in Figure S2 and Fig. 8, the pressure fluctuation trend of the suction surface under different working conditions was basically the same, and the pressure curve in the time-domain graph was relatively smooth, indicating that the change in the pressure of the suction surface was less affected by flow velocity, which was related to the structural characteristics of the suction surface and the flow characteristics of the fluid in this region. Figure 8 shows the pressure spectra at different monitoring points on the suction surface of the impeller under different working conditions. It can be seen that the pressure fluctuations on the suction surface are mainly concentrated at the leading edge of the blade, which is due to the intense impact of the fluid at the leading edge of the blade and the change in the flow state. The positional change of the pressure value on the suction surface in the streamline direction was the same as that on the pressure surface, indicating that the overall structure of the impeller played a certain guiding role in the pressure distribution. In the span direction, the pressure fluctuations were concentrated at the trailing edge of the blade near the hub, peaking at 5*f_r_, which was attributed to the complex fluid flow in this region and the rotational effect of the impeller.

**Fig. 9:**
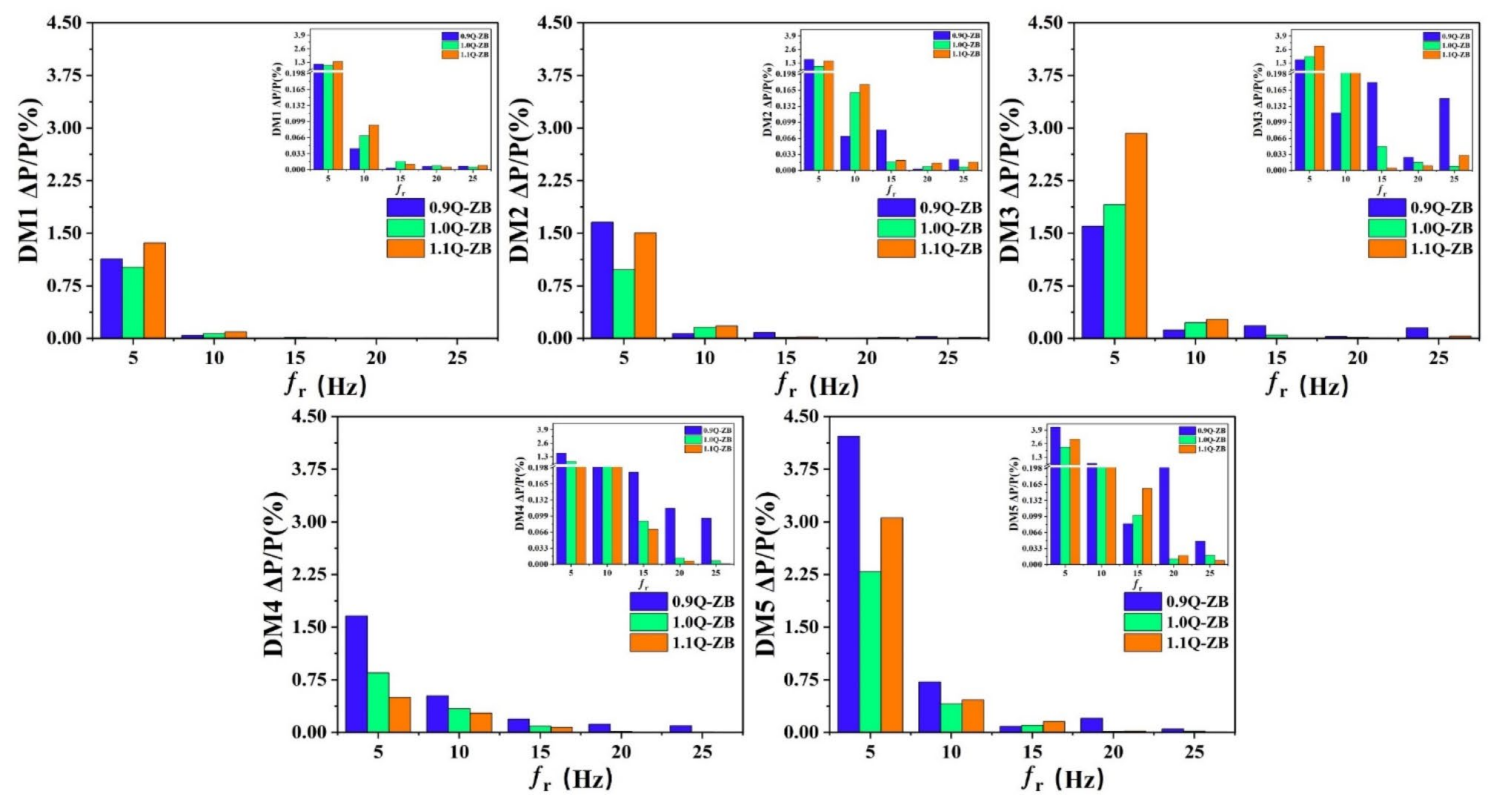
Spectra of pressure at different monitoring points on the inlet and outlet end faces of impeller.

Finally, the inlet and outlet end faces of the impeller were analysed. As shown in Figure S3 and Fig. 9, at the mid-pitch, the pressure fluctuation at the outlet end face was the smallest, while that at the inlet end face was the largest. Figure 9 shows the pressure spectra at different monitoring points on the inlet and outlet end faces of the impeller under different working conditions. It can be seen that at the design flow rate, the pressure fluctuations at the inlet and outlet end faces of the impeller are relatively small, indicating that the performance of the impeller is more stable at the design flow rate. When the flow rate met the design requirements, the internal flow within the impeller was more stable, and the pressure was more uniformly distributed, hence reducing the pressure fluctuations.

#### Pressure fluctuation of clearance flow

In the high-specific speed-operation process of the pump, the pressure fluctuation of the clearance flow also exerted a significant impact on the performance of the pump. The time-domain and frequency-domain characteristics of the pressure at the tip clearance of impeller are shown in Figs. S4 and S5, which reveal the mechanism of its impact on the performance of the pump.

As shown in Figure S4, the pressure at monitoring point P1 located at the leading edge of the impeller was the greatest at the design flow of 1.0Q_d_, indicating that pressure was concentrated at the leading edge of the blade in the tip clearance. This was closely related to the rotational speed of impeller, the inflow angle of fluid, and the geometrical shape of tip clearance. In case that fluid entered the impeller at a certain speed and angle, pressure in this region would increase due to the special structure of the leading edge and the inertial effect of the fluid. By comparing the pressure of the impeller under three different working conditions, the pressure at the design flow of 0.9Q_d_ was generally greater than that under the other two working conditions. In particular, the pressure at monitoring point P1 was the largest, which further confirms that the pressure at the gap of the leading edge of the blade was the largest. When the design flow was close to 0.9Q_d_, the flow velocity and flow distribution of the fluid caused the fluid at the leading edge of the impeller to suffer greater obstruction and extrusion, resulting in an increase in pressure.

To better understand the frequency characteristics of pressure fluctuations, the pressure spectrogram of different monitoring points in the impeller gap within one cycle was plotted, as shown in (Fig. S5). It can be seen from the figure that the peak amplitudes of the pressure fluctuations at each monitoring point were mainly concentrated at 5 and 10*f_r_, with its largest value at monitoring point P4, indicating that the pressure fluctuation at the top clearance of the impeller near the trailing edge was more intense. This was closely related to the internal flow field structure of the impeller and the formation and development of vortices. The pressure fluctuation at the trailing edge was even more significant because of the interaction between the outflow of the fluid and vortices.

In addition, by comparing the peak changes displayed in Fig. S5, there were significantly less peaks at the design flow of 1.0Q_d_ than at the other two design flow because the pressure pulsation in the impeller clearance was relatively small under such condition, and the performance of the pump was more stable. Under the design flow, the flow state of the fluid was more in line with the design requirements of the impeller, with smoother flow and reduced pressure fluctuations. By observing the trend of the curves in Fig. S5, we found that the trends of the three curves TM, MM, and SM were highly similar, indicating that the distribution trend of pressure fluctuations at different tip clearance heights basically remained unchanged at different clearance heights. While the variation in tip clearance height had little impact on the distribution of pressure fluctuations, the aforementioned analysis clearly showed that the pressure fluctuation at the tip clearance of the leading edge of the impeller was the most significant. This finding reveals that the flow pattern around the tip clearance of the impeller had a significant influence on the operating stability of the pump. Especially at high speeds, the pressure fluctuations at the tip clearance of the impeller resulted in vibration and noise, thereby affecting the performance and reliability of the pump.

### Hydrodynamic performance

In order to further explore the mechanical properties of the new impeller pump under different operating conditions, the pump vanes were mechanically monitored in the X, Y, and Z directions to obtain the values of the axial force Fa and the radial force Fr of the impeller, of which the former could be directly measured while the latter could be calculated by:

10 $$F_{r} = \sqrt {F_{x} ^{{\;2}} + F_{y} ^{{\;2}} }$$

where F_x_ denotes the force in the X direction and F_y_ denotes the force in the Y direction.

**Fig. 10:**
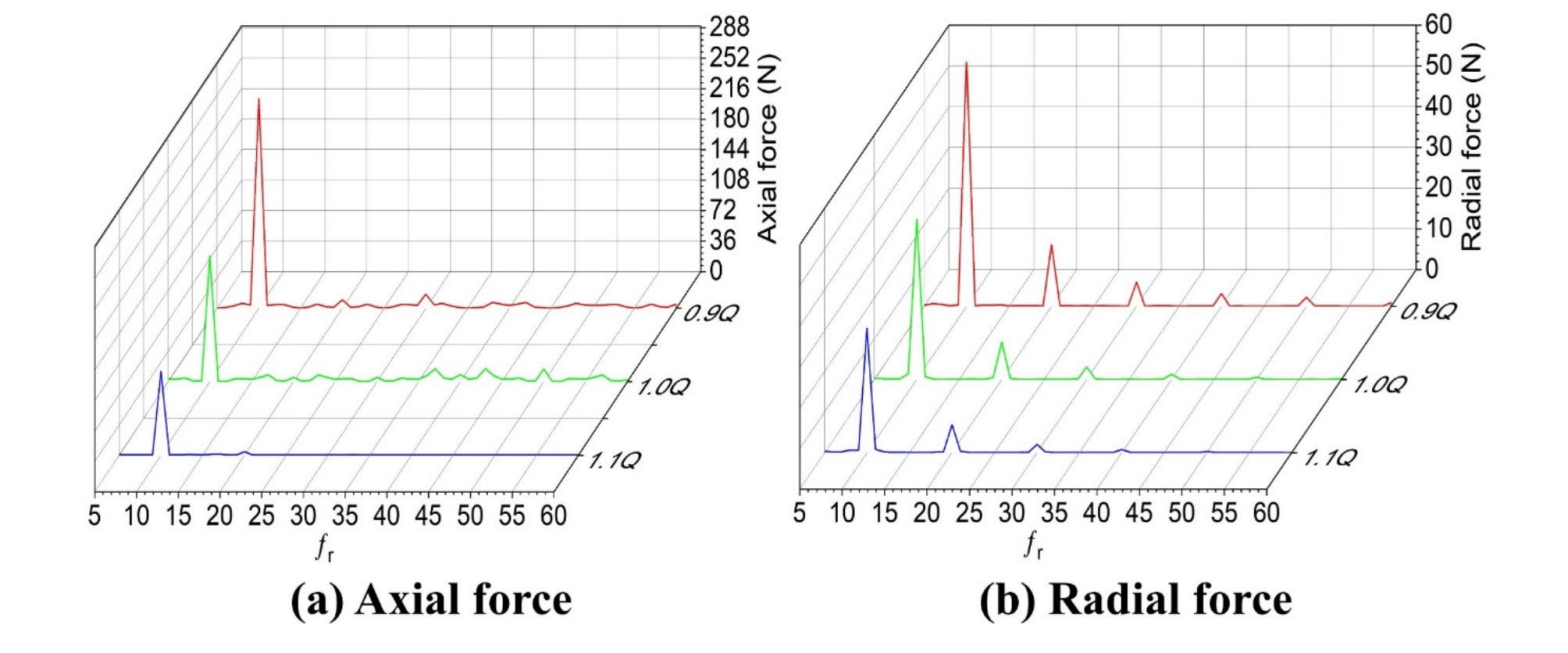
Spectrograms of axial and radial forces on the impeller.

As shown in Fig. S6 and Fig. 10, the axial force exhibited a cyclic changing tendency under three working conditions. Among them, the axial force at the design flow of 0.9Q_d_ was the most notable, with a maximum value of 22,900 N, which was closely related to the flow rate and the rotational speed of the impeller, as well as the flow characteristics of the fluid. Under such working condition, the fluid flow inside the impeller was significantly impeded because of a relatively small flow rate, resulting in a relatively large axial force. In contrast, the axial forces at the design flow of 1.0 and 1.1Q_d_ successively decreased, which reflects the influence of flow rate on the axial force. With the increase of flow rate, the fluid flow within impeller became smoother, which reduced resistance and axial force accordingly.

Figure 10a further reveals the spectral characteristics of the axial force. The axial force showed a relatively large amplitude at 10*fr under all the working conditions, which indicates that the specific multiple of the rotational frequency was associated with the fluctuation of the axial force during the rotation of the impeller, which was caused by the structure of the impeller and the resonance of the fluid. It is particularly notable that the amplitude fluctuated more obviously at the design flow of 0.9Q_d_, while less significantly at the design flow of 1.0Q_d_, showing a more stable performance. This was because the flow was more unstable at the design flow of 0.9Q_d_, resulting in an intensified fluctuation of the axial force, while that at the design flow of 1.0Q_d_ was relatively stable. As a result, the axial force fluctuated less significantly.

Furthermore, Fig. S6b shows the time-domain change of the radial force within one cycle. The radial force is generally relatively small under the three operating conditions. Specifically, the radial force is the largest at 163 N in the case of 0.9Q_d_, and decreases successively in the cases of 1.0 and 1.1Q_d_. This changing trend is similar to that of the axial force, which is also due to the different flow states of the fluid and the force-bearing situation of the impeller under different working conditions. Figure 10b provides the spectral analysis of the radial force. The amplitude of the radial force also occurs at the rotational frequency of a multiple of 10 and decreases as the rotational frequency increases. This once again indicates that there is a certain pattern between the rotational frequency of the impeller and the fluctuation of the radial force, which is related to the rotational dynamics of the impeller and the interaction of the fluid.

By comparing the mechanical properties under three different working conditions, it can be seen that under the design flow rate of 1.0Q_d_, the new impeller pump exhibits relatively large axial and radial forces, while the amplitude fluctuation is relatively small, and thus shows excellent mechanical performance. This is because at the design flow rate, the structure of the impeller and the flow of the fluid can achieve a better match, making the force on the impeller more uniform and stable, thereby improving the mechanical performance. However, further analysis is needed to understand how these mechanical properties affect the operational stability and reliability of the pump, and how they can be optimized to enhance the performance of the pump.

### Internal flow field characteristics

The fluid in the pump acquired kinetic energy by interacting with the pump blades, generating specific pressure and velocity. Optimizing this pressure-velocity field is crucial for efficient fluid transmission, as it reduces energy loss, improves pump efficiency, and maintains consistent performance in different application scenarios.

**Fig. 11:**
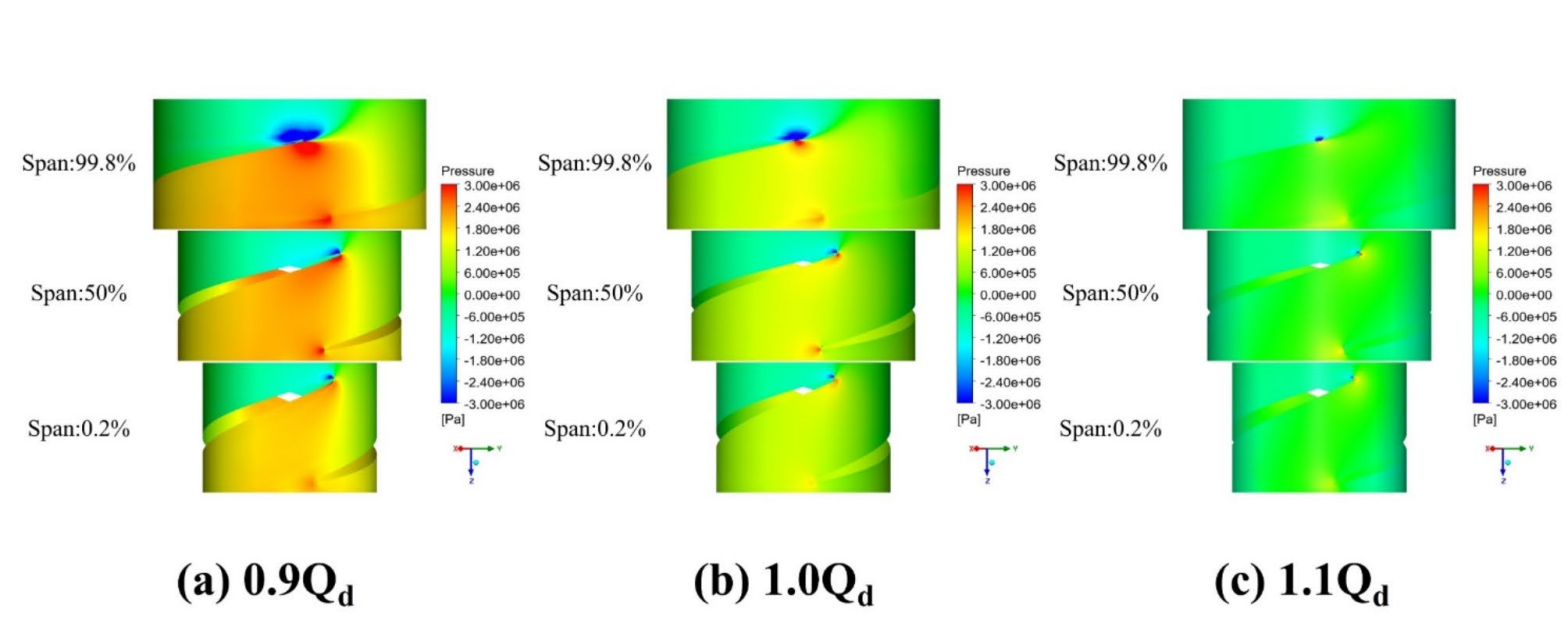
Pressure distribution on the blades at different flow rates.

As shown in Fig. 11, at different blade heights with different flow rates, the pressure on the leading edge of the blade was the largest and mainly concentrated at 99.8% of the blade height. This was closely related to the impact angle and speed of the fluid during its entrance into the blade, as well as the geometry of the blades. When the fluid affected the leading edge of the blades at a certain speed and angle, pressure concentration emerged due to the relatively small contact surface and the concentrated action of the kinetic energy of the fluid in this area. While the trailing edge also experienced higher pressure, the pressure was more unevenly distributed in this region than that at the leading edge because the flow state at the trailing edge became more complex after the fluid accelerated and pressure changed at the leading edge and the pressure distribution was relatively dispersed due to the existence of vortices and backflow. These findings indicate that the pressure of the flow field at the blade tip clearance changed more significantly and was mainly concentrated at the clearance of the leading edge, further reflecting the flow characteristics of the fluid at the blade tip clearance. The leading edge was the first to be contacted by the fluid and the existence of clearance led to the changes in the flow resistance, making the pressure in this region even more concentrated.

By comparing the pressure distributions at different blade heights, we discovered that with the decrease of blade height, the flow field pressure decreased, because reduced blade height affected the fluid flow channel and path, thus increasing the energy loss and reducing pressure. With the increase of the flow rate, the pressure difference on both sides of the blade flow field decreased, and the pressure concentration at the leading edge of the blade was weakened, which indicates that the change of the flow rate on both sides of the blade had a relatively large influence on the flow field. The increased flow rate changed the flow speed and direction of the fluid, making the pressure distribution in the flow field more uniformly distributed, thereby reducing the pressure difference while alleviating the pressure concentration at the leading edge of the blade.

### Vortex characteristics

The vortex characteristics of the pump were examined. In the field of vortex identification, vortex is usually regarded as the region where vorticity reaches its peak or maximum. Currently, several methods have been proposed for identifying vorticity, such as the Q criterion, the Δ-criterion and the λ_2_-criterion models, which are all based on a regional criterion and have Galilean invariance. Among them, Hunt et al.^32^ proposed a Q vortex identification method based on the strain rate tensor S_ij_ and vortices tensor Ω_ij_. Subsequently, Chong et al.^33^ further proposed the Δ criterion, while Hussain et al.^34^ summarized previous studies and proposed a new vortex identification criterion, namely the λ_2_ criterion. All three vortex criteria possess Galilean invariance and are widely used in the field of vortex identification. In addition, vortices strength^35^ and strain acceleration tensor^36^ have also been used for vortex identification. The flow in the tip clearance of the pump formed a series of vortices, mainly including two typical tip sweep vortices and tip leakage vortices.

**Fig. 12:**
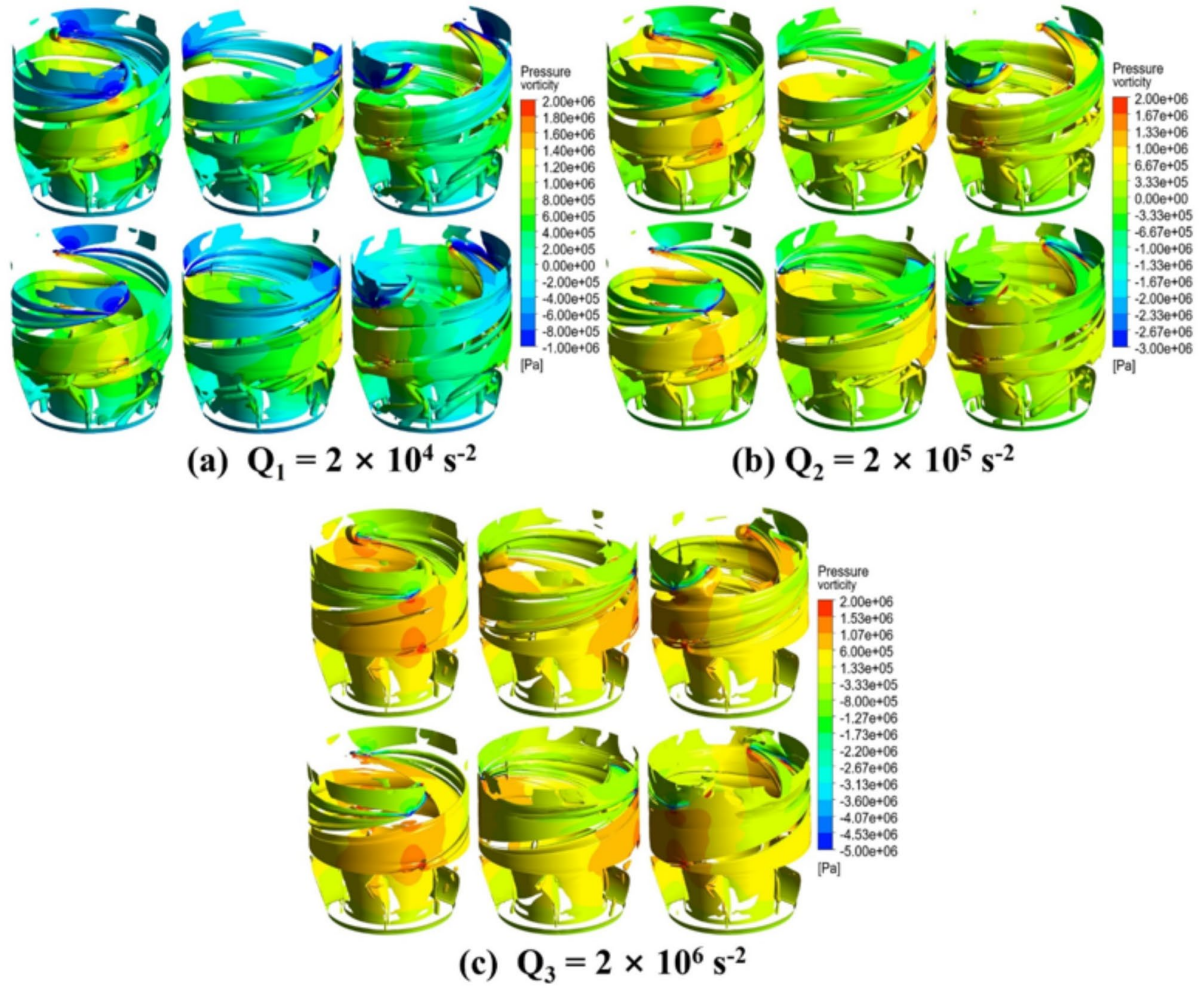
Pressure distributions on vortices iso-surfaces in the impeller and guide vane domains for three different Q criteria at the flow rate of 1.0X times.

To study the performance and dynamic changes of vortex in the new impeller, three vortex equivalent surfaces with different Q criteria (Q_1_ = 2 × 10^4^ s^− 2^, Q_2_ = 2 × 10^5^ s^− 2^, and Q_3_ = 2 × 10^6^ s ^− 2^) were selected at the design flow rate of 1.0Qd. As shown in Fig. 12, the jet of the new impeller pump had a rotational cycle. The cloud maps of the impeller and guide vane areas were colored according to the pressure on the vortex equivalent surfaces. By comparing the vortex changes under the three criteria, we found that the vortex in the impeller increased with the Q value, because the higher the Q value, the stronger the vortex intensity criterion, and the weaker the vortex structure. Meanwhile, the vortices in the guide vane area decreased as the guide vane had an inhibitory or dispersing effect on the vortex.

Figure 12a–c detail the change in the pressure of the impeller vortex within one rotational cycle. It can be observed from the Figure that the pressure distribution at the leading edge of the blade vortex was relatively large due to the pressure concentration caused by the tip clearance, which restricted the fluid flow and converted kinetic energy into pressure energy. In contrast, the pressure was more uniformly distributed on the vortex in the middle of the blade and gradually decreased because of the relatively stable fluid flow and the balance between the conversion and distribution of pressure energy. By comparing the pressure distribution shown in Fig. 12, the vortex equivalent surface with Q_2_ = 2 × 10^5^ s^− 2^ is more effective in reflecting the pressure distribution in the impeller and guide vane domains, which was attributed to its strong ability to capture the vortex structure with a significant influence and accurately reflect the key characteristics of pressure distribution.

To further investigate the influence of different flow velocities on the vortex, the vortex equivalent surface at Q_2_ = 2 × 10^5^ s^− 2^ was selected for pressure distribution analysis. As shown in Fig. S7, the vortices in the impeller and guide vane domains gradually decreased with the increase in the flow velocity because of the increased inertial force of fluid, thus weakening the effect on the structure of vortex. The pressure distribution on the isotropic surface of the vortex remained stable. The vortex entered the guide vane through the impeller, being subject to the truncation and upward stretching effect of the leading edge of the guide vane and thus changing the flow path and form of the vortex. The vortex stretched in the axial direction when entering the guide vane due to relatively high local velocity. The high-specific-speed vortex accelerated the flow near the pressure side, generating a back pressure effect under the interaction between energy transfer and fluid and ultimately resulting in the change of pressure and flow adjustment.

While guide vane can slightly change the shape of the vortex, the size of vortex basically remained unchanged, which indicates that the main role of the guide vane was to make local adjustments to the morphology of vortex with limited impact on the overall scale and intensity of the vortex. When the vortex flowed out of the guide vane, its intensity greatly changed under the combined effect of low-speed flow and the shear layer of pipe, resulting in energy loss and structural changes.

As can be seen from vortex evolution process, pressure and velocity distribution and wake energy projection, decreased open water efficiency was caused by not only the weakened influence of impeller on flow, but also the negligible influence of guide vane on the recovery the flow at tip clearance. This finding reveals the mechanism of the interaction between the impeller and the guide vane in the vortex formation and evolution process, which provides an important theoretical basis for optimising the design of the impeller and the guide vane and improving the performance of the pump.

## Conclusions

Combining numerical simulations and experiments, this paper analysed the unsteady flow in a high-specific-speed axial impeller with a small aspect ratio and obtained the unsteady pressure fluctuation, hydrodynamic characteristics, flow field, and the evolution mechanism of vortices under various operating conditions. The main conclusions are listed as follows:

1. In the main flow field, the pressure on the pressure surface at 0.9Q_d_ was the highest but fluctuated irregularly, causing significant vibrations and noises. In contrast, the pressure change on the suction surface was minimally influenced by flow velocity, fluctuating most obviously at the leading edge. At the inlet and outlet end faces of the impeller, the outlet pressure at the mid-pitch was the lowest, while the inlet pressure fluctuated most significantly. At the clearance flow of 1.0Q_d_, the pressure at the monitoring points of the leading edge was the highest. The peak amplitudes of pressure fluctuations mainly emerged at 5 f_r_ and 10 f_r_, with its maximum value at the tip clearance near the trailing edge. At 1.0Q_d_, there were fewer peaks, and the pressure fluctuation patterns remained consistent across different tip clearance heights, with the most severe fluctuation at the leading edge. This result reveals the intricate relationship between pressure fluctuation and the geometric shape of the impeller, rotational speed, fluid flow characteristics, and working conditions, as well as their influence on the performance of pump.
2. The axial force demonstrated a cyclic variation trend under the three working conditions, with the largest value at 0.9Q_d_, successively followed by those at 1.0Q_d_ and 1.1Q_d_. The amplitude of the axial force was relatively large at the rotational frequency of 10 times, with obvious amplitude fluctuation at 0.9Q_d_ and relatively small fluctuation at 1.0Q_d_, indicating more stable performance. The radial force was the largest at 0.9Q_d_, successively followed by those at 1.0Q_d_ and 1.1Q_d_. The amplitude of the radial force also occurred at 10 times the rotation frequency and decreased with the increase of the rotational frequency. At the design flow rate of 1.0Q_d_, the new impeller pump demonstrates excellent mechanical performance.
3. Use different vortex structure Q criteria to analysis, the result is consistent. By comparing the changes in vortices under various Q criteria, an increase in the Q value led to a gradual increase in the vortices in the impeller, while vortices in the guide vane decreased correspondingly. With the rise of flow velocity, the vortices in both the impeller and guide vane domains gradually decreased, and the pressure distribution on the isotropic vortex surface remained stable. The guide vane locally adjusted the morphology of vortices without significantly affecting its size. Meanwhile, the vortex intensity changed significantly when vortices exited from the guide vane. This provides valuable insights into the formation and evolution mechanism of vortices, laying an important foundation for optimising the design of the impeller and the guide vane.

## Electronic supplementary material

Below is the link to the electronic supplementary material.

Supplementary Material 1

## Data Availability

Availability of Data and MaterialsThe datasets used and analysed in this study are available upon reasonable request. Anyone who needs to obtain the data, please contact the corresponding author of this study [Fangming Zhou], and the contact email is [xiaomagehai@126.com]. After receiving the request, the corresponding author will evaluate and provide the corresponding datasets based on the specific situation.
