# Supplementary material for: Research on the unsteady flow characteristics of high specific speed axial flow impellers with small aspect ratio and double blades
Source: Sci Rep. 2025 Mar 10;15:8268. doi: 10.1038/s41598-025-85597-9 (PMC11893745; doi:10.1038/s41598-025-85597-9)
Supplement: Supplementary file 1 — Supplementary Material 1 [file 41598_2025_85597_MOESM1_ESM.pdf]

Supplementary Information

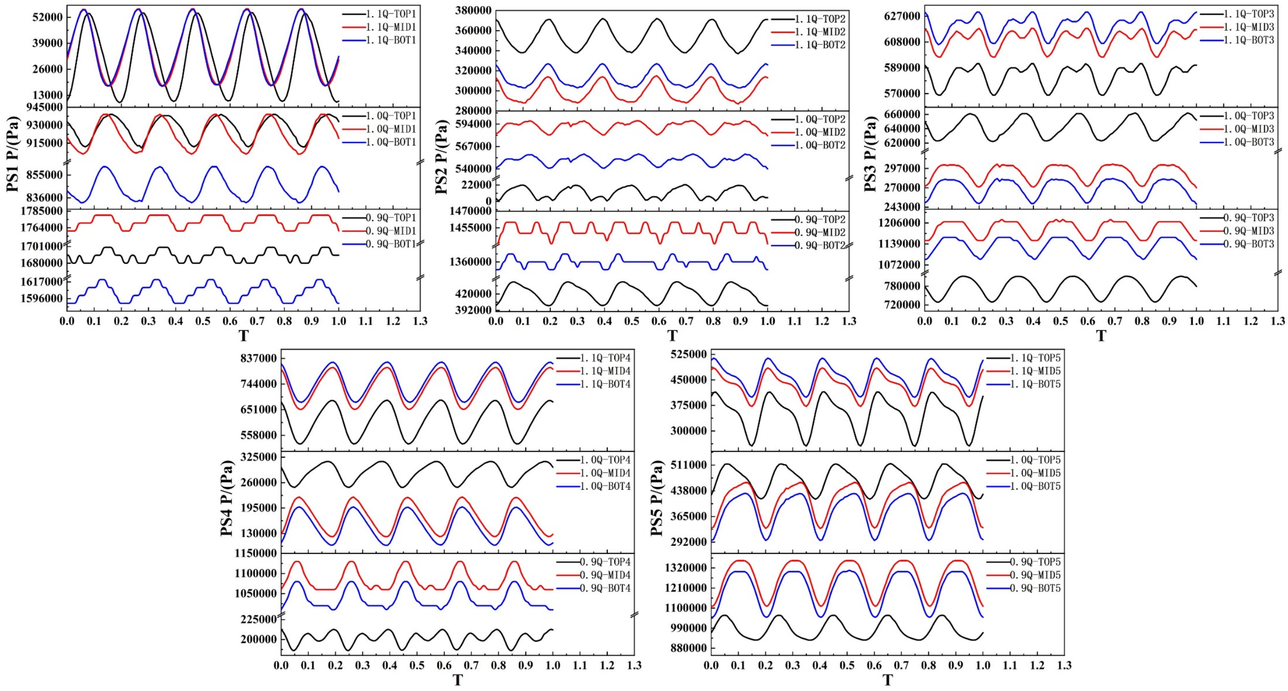

Fig. S1 Time-domain plot of pressure at different monitoring points on the pressure surface of impeller

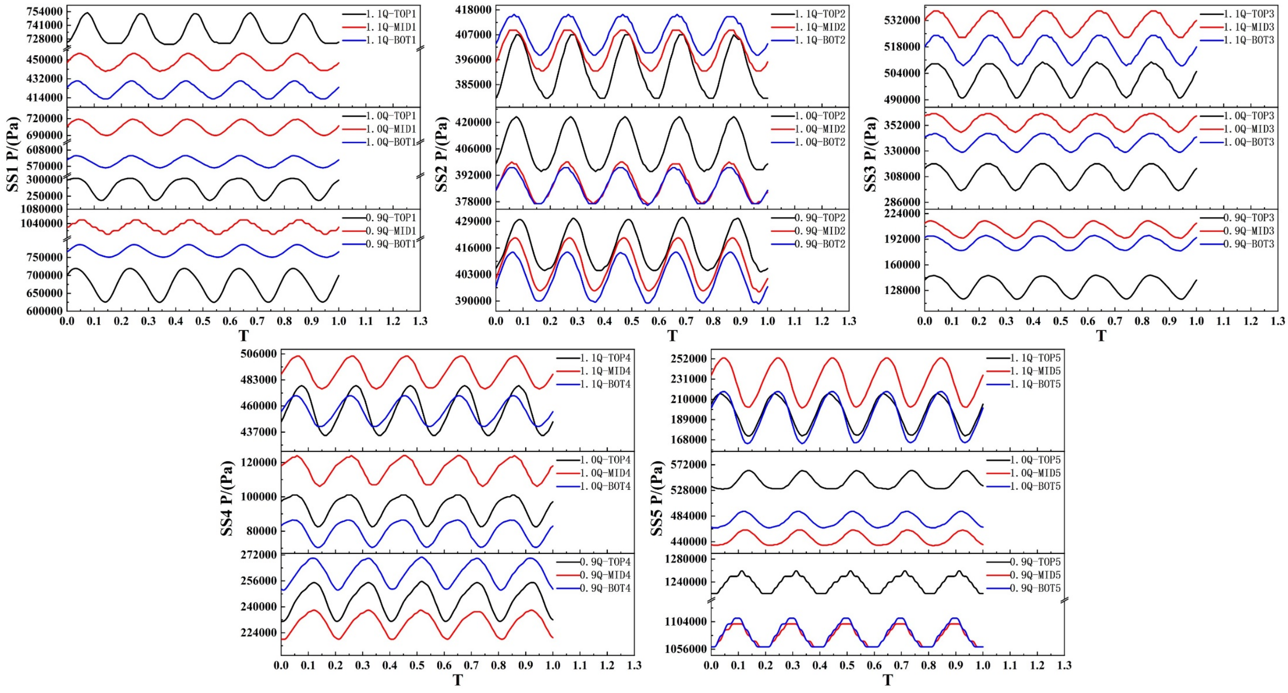

Fig. S2 Time-domain plot of pressure at different monitoring points on the suction surface of impeller

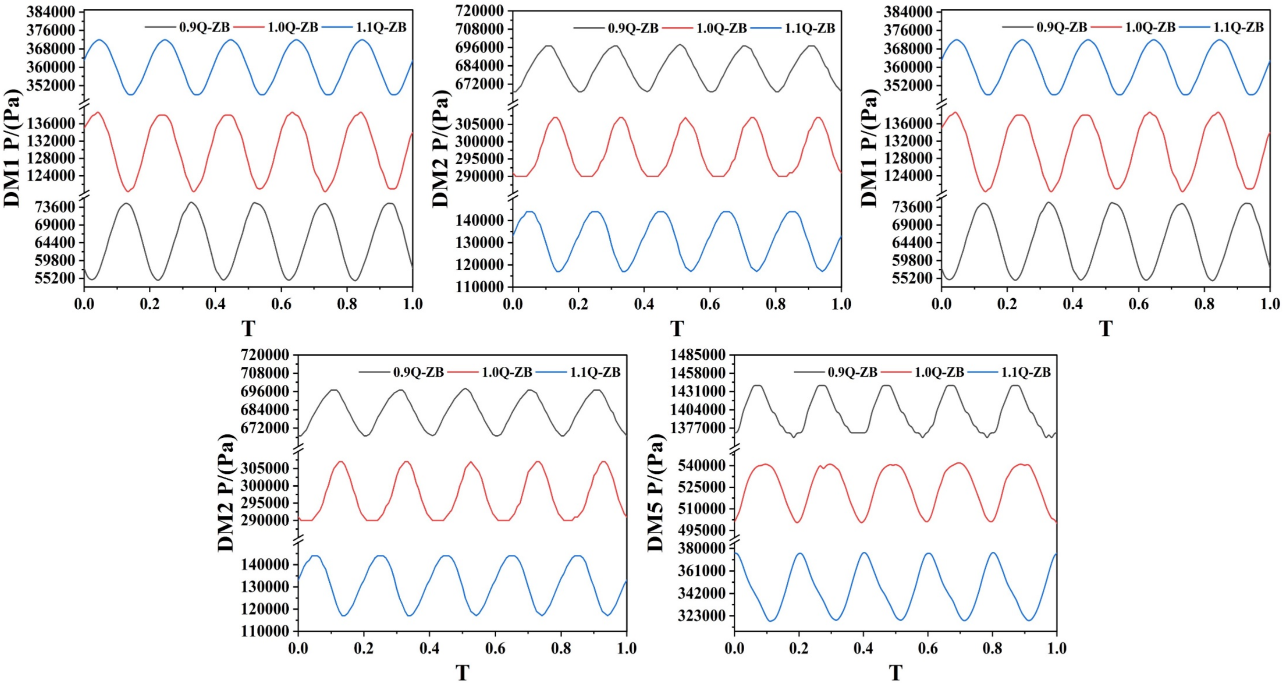

Fig. S3 Time-domain plot of pressure at different monitoring points on the inlet and outlet end faces of impeller

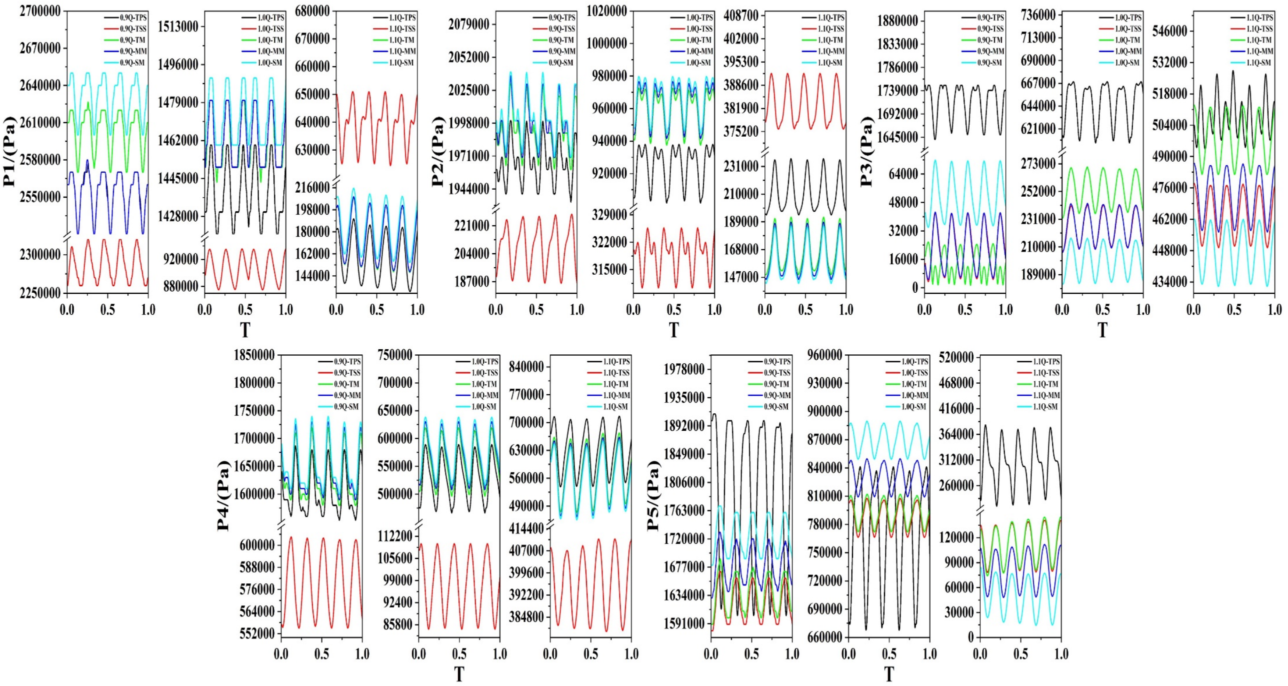

Fig. S4 Time-domain plot of pressure at different monitoring points of impeller clearance

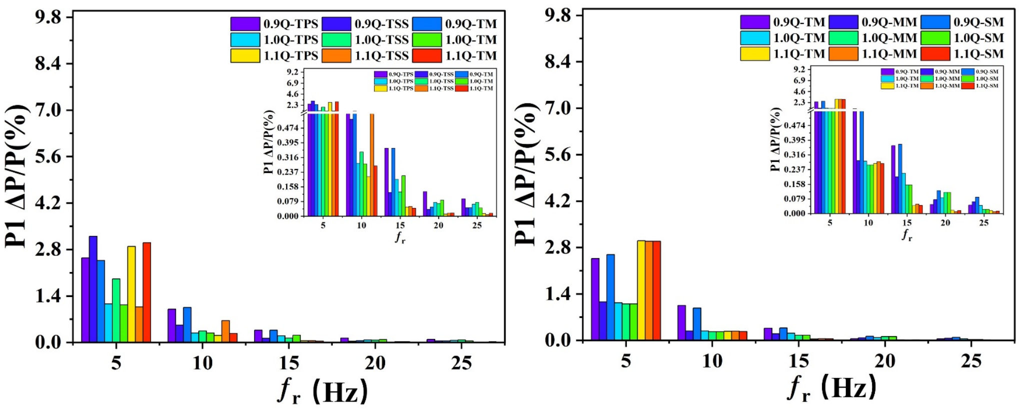

(a) P1 monitoring point

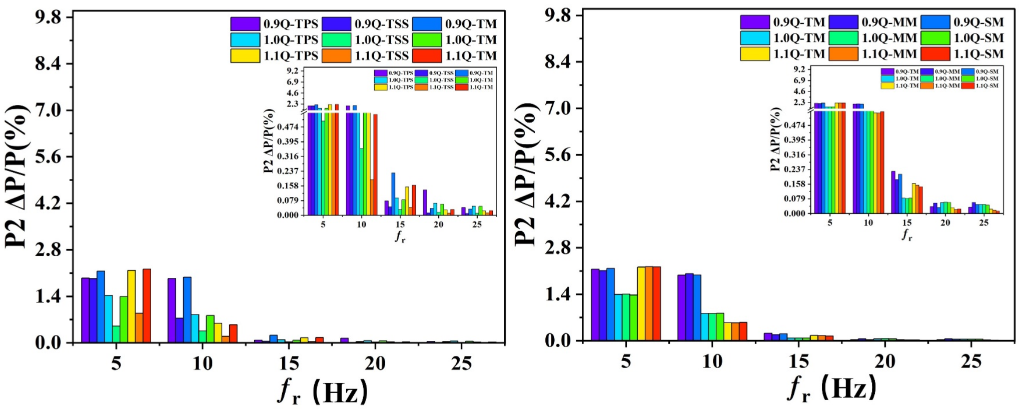

(b) P2 monitoring point

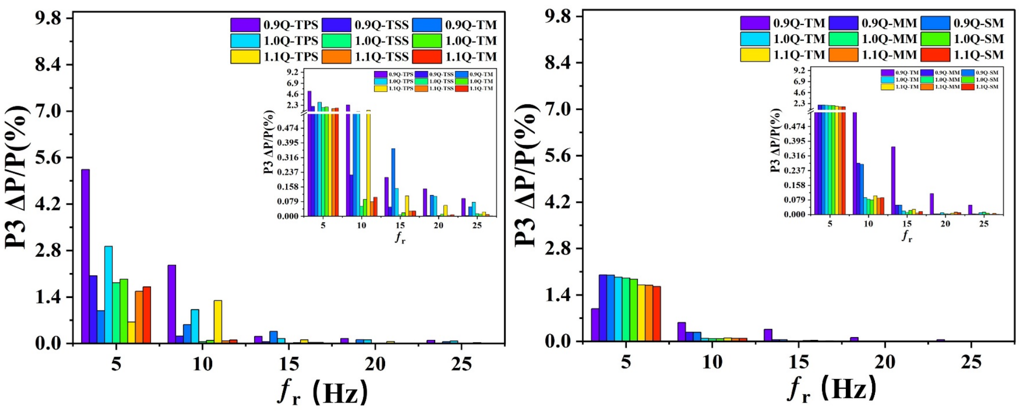

(c) P3 monitoring point

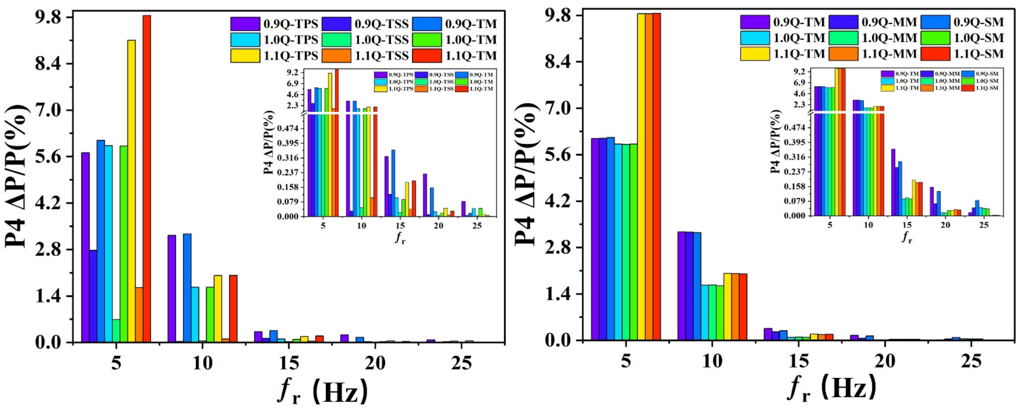

(d) P4 monitoring point

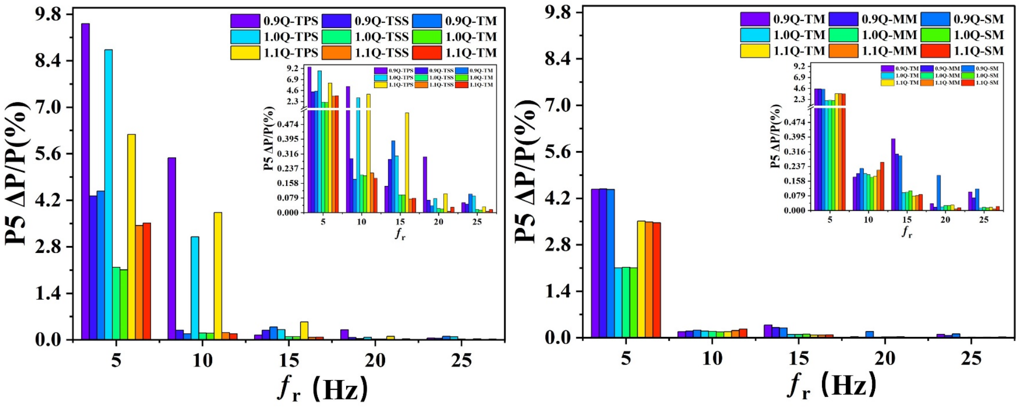

(e) P5 monitoring point

Fig. S5 Spectrograms of pressure at different monitoring points of impeller clearance

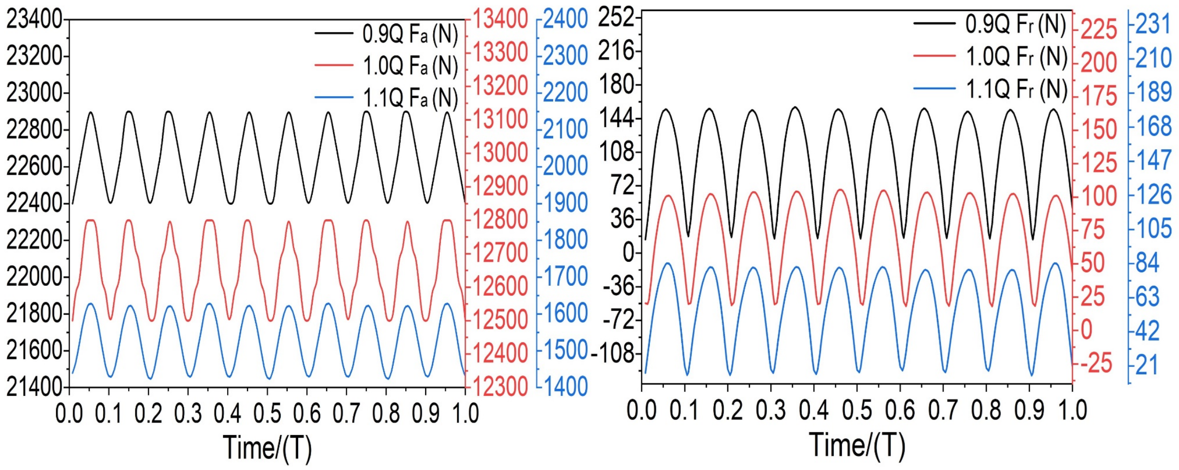

(a) Axial force **(b) Radial force**

Fig. S6 Time-domain diagrams of axial and radial forces on the impeller

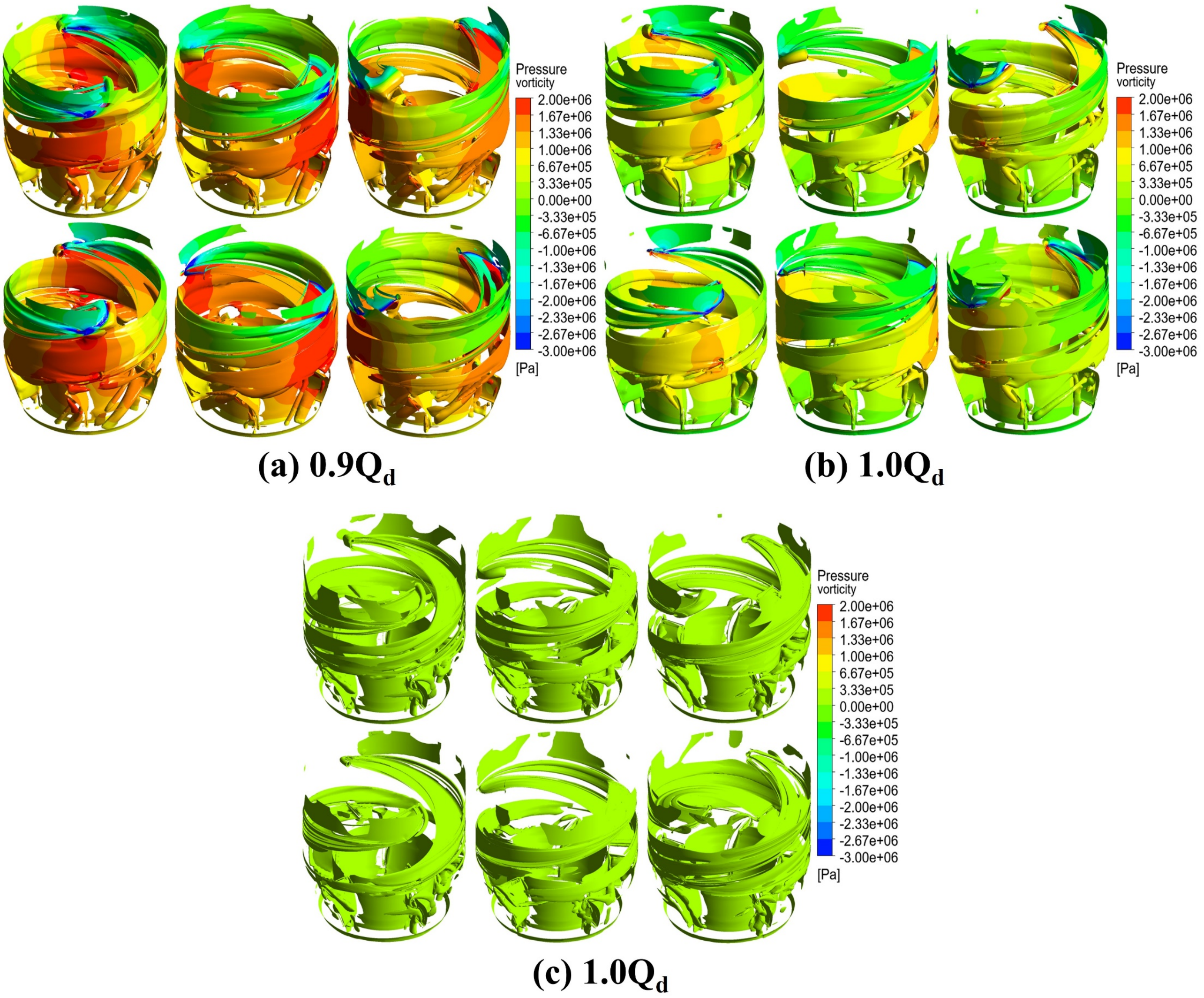

Fig. S7 Pressure distribution on vortices iso-surfaces for $Q_2 = 2 \times 10^5$ s^{-2} at different flow rates
